# Long-term results of peritoneovenous shunt implantation for refractory ascites in patients with alcoholic liver cirrhosis: single-center retrospective study of 348 patients

**DOI:** 10.1136/bmjsit-2025-000433

**Published:** 2026-01-22

**Authors:** Krisztina Fekete, Péter Kupcsulik

**Affiliations:** Department of Surgery, Transplantation and Gastroenterology, Semmelweis Egyetem, Budapest, Hungary

**Keywords:** Cohort Study, Patient Outcome Assessment, Devices

## Abstract

**Introduction:**

Despite its reasonable pathophysiological basis, peritoneovenous shunt (PVS) implantation currently plays a very limited role in managing patients with refractory ascites. On the other hand, we have 20 years of experience using PVSs. The aim of this study was to evaluate the clinical efficacy and long-term outcomes of PVS implantation for the treatment of refractory ascites caused by alcoholic liver cirrhosis.

**Methods:**

We conducted a retrospective review of 348 consecutive patients who underwent PVS placement. Survival data were compared in subgroups according to: (1) severity of liver cirrhosis; (2) occurrence of shunt occlusion; and (3) patients who had contraindications for liver transplantation (LT).

**Results:**

The 1-year and 5-year survival rates for the entire study population were 63% and 23%, respectively. In the shunt occlusion group, the survival rate was comparable with that of the control group: 1-year survival rate was 73%, and 5-year survival rate was 26%. In the strictly selected population where LT was contraindicated, the 1-year and 5-year survival rates were 53% and 20%, respectively.

**Conclusion:**

For a well-selected group of patients, PVS implantation is a favorable choice. However, long-term survival data do not justify the highly restricted role of PVS in treatment guidelines.

**WHAT IS ALREADY KNOWN ON THIS TOPIC**

- Despite its reasonable pathophysiological basis, peritoneovenous shunt (PVS) implantation currently plays a very limited role in managing patients with refractory ascites. The frequency of shunt occlusion is the most important argument against PVS implantation.

**WHAT THIS STUDY ADDS**

- In our study, PVS occlusion was not considered as a failure of the therapeutic modality because we can treat it with a low-risk second surgery. 1-year survival rate in our study population was 63% and 5-year survival rate was 23%, which does not explain the highly restricted place of PVS in treatment guidelines.

**HOW THIS STUDY MIGHT AFFECT RESEARCH, PRACTICE, OR POLICY**

- PVS implantation may be a reasonable option in select patients, particularly where other options like transjugular intrahepatic portosystemic shunt or liver transplantation are not feasible.

## Introduction

Ascites is one of the major complications in cirrhosis. The onset of ascites marks a critical point in the progression of liver disease; it increases hospital admissions and costs of care.^1^ Over half of cirrhotic patients will develop ascites within 10 years of their diagnosis.^2–4^ Ascites becomes refractory to treatment with diuretics and sodium restriction in approximately 10% of patients.^5^ Once ascites becomes refractory to medical therapy, it means a poor prognosis; 50% of patients die within 6 months.^6^

The peritoneovenous shunt (PVS) facilitates the drainage of ascitic fluid from the high-pressure peritoneal cavity to the low-pressure central venous system. PVS produces a sustained intravascular volume expansion and suppresses the endogenous antinatriuretic systems, thereby increasing the renal response to diuretics.^7^

Despite the reasonable pathophysiological basis, according to the clinical guidelines, PVS implantation currently plays a very limited role in the management of patients with refractory ascites.^8 9^ At the Department of Surgery, Transplantation and Gastroenterology of Semmelweis University, however, we have a very good experience in the use of PVS.

We raised the question: should PVS implantation be avoided in the treatment of refractory ascites? We examined the clinical efficacy and long-term outcomes of the procedure over a 20-year period of frequent use of PVS implantation.

## Methods

### Procedure

LeVeen’s shunt consists of a fenestrated catheter placed in the abdominal cavity, connected through a one-way valve to another catheter, which reaches up to the neck in a subcutaneous tunnel and enters the subclavian or jugular vein. The flow in the shunt is maintained by pressure gradient. A variant of this is the Denver shunt, which has a manual pump that allows the patient to directly adjust the transfer of ascites to the bloodstream, thereby reducing the possibility of the device becoming occluded.^10^

In our department, PVS implantation was indicated for patients with refractory ascites according to the International Ascites Club definition,^11 12^ and the patients’ quality of life had deteriorated due to the ascites.

The contraindications were: (1) evidence of infected ascites; (2) severe heart failure; (3) the presence of hepatorenal syndrome; (4) total serum bilirubin value more than 50 μmol/L; and (5) coagulation disorder characterized by prothrombin time ratio lower than 50% and international normalized ratio (INR) greater than 2.2.

A diagnostic paracentesis was performed to rule out infected ascites and peritoneal carcinosis. Cardiac output was assessed using echocardiography. Upper gastrointestinal panendoscopy was required to reduce bleeding complications. In the event of grades 3–4 esophageal varices, sclerotherapy was performed 2 weeks prior to shunt implantation.

The operations were performed under general anesthesia. Following a right subcostal minilaparotomy, the abdominal limb of PVS was implanted into the peritoneal cavity, the pumpable valve of the Denver shunt was placed over the chest wall, usually located subcutaneously over the 11th–12th rib. The vascular limb of the PVS was introduced through a lateral incision into the jugular vein, and the tip was placed at 2 cm from the atriocaval junction. The final location of the tip of the vascular limb was evaluated using fluoroscopy.

During the surgery, 4000–5000 mL of the ascites fluid was removed and replaced with 2000 mL body-temperature Ringer’s lactate solution to reduce the incidence of postoperative coagulopathy. Preoperatively, prophylactic broad-spectrum antibiotics were administered and continued for up to 48 hours.

At the end of the operation, we measured the clotting time, and we started anticoagulant treatment with intravenous unfractionated heparin. During the immediate postoperative period, we continued heparin treatment with activated partial thromboplastin time control. On the third to fifth postoperative day, we introduced oral anticoagulation treatment with acenocoumarol controlled by INR, and the target range was INR 3.0–3.5. The patients learned special physiotherapy techniques to improve fluid flow using their abdominal muscles.

All patients underwent regular hepatological examination every 3 months. Their abdominal circumference and laboratory tests, including liver enzymes, kidney function, and INR, were monitored. If a repeated increase was observed in abdominal circumference, a surgical examination was performed. The diagnosis of shunt occlusion was confirmed by fluoroscopy. In case of shunt occlusion without septic signs, we performed reoperation and shunt desobliteration.

### Setting and population

The aim of this retrospective study was to evaluate the clinical efficacy and long-term outcomes of PVS implantation for the treatment of refractory ascites due to alcoholic liver cirrhosis. We were interested in accurate long-term survival data.

We retrospectively reviewed 348 consecutive patients with refractory ascites, who underwent PVS placement between January 1995 and December 2015, at the Semmelweis University, First Dept. of Surgery.

All patients were diagnosed with refractory ascites according to the International Ascites Club definition.^11 12^ Ascites was secondary to alcoholic cirrhosis in all cases. Patients with viral hepatitis, hepatocellular carcinoma, or other malignancies were ruled out. None of the patients in the trial underwent transplantation, which was available within narrow limits in Hungary before the early 2000s.^13^

We studied 246 men and 102 women. Mean age of the patients was 55 years (SD: 10 years). Liver cirrhosis was classified as Child B in 245 patients and Child C in 103; no patient was Child A. Model for end-stage liver disease (MELD) score^14^ was calculated retrospectively, and mean MELD score was 15 (table 1).

**Table 1:** Patient characteristics

	Gender		Age (year)	
	Male	Female	≤ 59	≥ 60
Number of patients	246	102	244	104
%	71%	29%	70%	30%

	Child-Pugh score	
	Child-Pugh B	Child-Pugh C
Number of patients	245	103
%	70%	30%

	MELD score			
	≤9	10–19	20–29	30–39
Number of patients	79	179	90	0
%	23%	51%	26%	0

MELD, model for end-stage liver disease.

During the preoperative examination, grades 3–4 esophageal varices were found in 42 patients, who received sclerotherapy 2 weeks prior to the operation.

In case of shunt occlusion, our protocol was to indicate corrective surgery. Shunt occlusion was observed in 128 patients during the follow-up period. Of these patients, 112 underwent corrective surgery and shunt desobliteration was performed.

As in 2006, routine transjugular intrahepatic portosystemic shunt (TIPS) insertion became available; it became the first treatment of choice in refractory ascites for patients who were eligible for liver transplantation (LT).^9^ After 2006, the number of PVS implantations halved, and all patients had a contraindication for LT; the mean age of the patient population was higher (65 years, SD: 10 years) and most patients had encephalopathy in their medical history. Child-Pugh and MELD scores were also higher than before.

### Statistical analysis

The overall survival curves were estimated using the Kaplan–Meier method; selected variables were compared using a two-sided log-rank test and Cox proportional hazards model. A p value of<0.05 was considered as the statistically significant threshold. IBM SPSS Statistics V.27.0 was used for all statistical analyses.

## Result

Based on the Kaplan–Meier method, the mean survival time of the entire study population was recorded at 43 months (95% CI 37 to 49 months), with a median survival time of 25 months (95% CI 21 to 30 months).

Mean survival time was found to be 53 months (95% CI 46 to 61 months) in Group Child-Pugh B and 20 months (95% CI 13 to 26 months) in Group C. Median survival time was 34 months (95% CI 28 to 39 months) in Group Child-Pugh B and 7 months (95% CI 4 to 9 months) in Group C. The survival probability was significantly higher in Group Child-Pugh B (HR=2.33, 95% CI 1.83 to 2.95, p<0.001).

The mean survival time was 65 months (95% CI 51 to 79 months) in Group MELD<9, 48 months (95% CI 39 to 56 months) in Group MELD 10–19, and 15 months (95% CI 11 to 20 months) in Group MELD 20–29. The median survival time was 49 months (95% CI 34 to 64 months) in Group MELD<9, 28 months (95% CI 23 to 33 months) in Group MELD 10–19, and 6 months (95% CI 2 to 9 months) in Group MELD 20–29. We found a significant difference in survival probabilities between Groups MELD 10–19 and MELD 20–29 (HR=2.56, 95% CI 1.92 to 3.34, p<0.001).

1-year survival rate of the entire study population was 63% and 5-year survival rate was 23%. The worst prognosis of a higher Child-Pugh score was observed in 1-year and 5-year survival rates; in Group Child-Pugh B, it was 73% and 28%, and in Group Child-Pugh C, 40% and 11%, respectively. Similar data were found for groups with higher MELD scores. 1-year and 5-year survival rates were 72% and 38% in Group MELD<9, 69% and 24% in Group MELD 10–19, and 33% and 7% in Group MELD 20–29, respectively.

A total of 34 patients died within 2 months after surgery (9.7%). Operative mortality was significantly more frequent in patients with severe liver failure, in both Child-Pugh C and Group MELD 20–29. We also observed higher operative mortality in the subgroup after 2006 (table 2).

**Table 2:** Operative mortality

	Number of patients	Number of deaths	%
Total study population	348	34	10%
Shunt correction	128	15	12%
MELD score			
<9	79	4	5%
10–19	179	14	8%
20–29	90	16	18%
Child-Pugh score			
B	245	12	5%
C	103	22	21%
Before/after 2006			
Before 2006	266	21	8%
After 2006	82	13	15%

MELD, model for end-stage liver disease.

The complications within 2 months after surgery are summarized in table 3.

**Table 3:** Postoperative complication

	Number of patients	Number of deaths
Shunt-related complication		
Shunt infection	10	8
Ascites leakage	2	0
Incarcerated hernia	5	2
Hemostasis		
Gastrointestinal bleeding	6	4
DIC	1	1
Pulmonary embolism	1	1
Other		
Progression of liver failure	18	15
Pneumonia	4	1
Pancreatitis	1	1
Myocardial infarction	1	1
	49	34

DIC, disseminated intravascular coagulation.

During the long follow-up period, ascites completely disappeared in 27 cases (8.25%) with a notable improvement in liver function. These patients were abstinent from alcohol after shunt implantation, their shunt could be removed, and the ascites did not recur thereafter.

Shunt occlusion was observed in 138 patients. In half of these cases, the occlusion occurred within 6 months of shunt implantation. 128 patients underwent reoperation and shunt desobliteration was performed.

We examined the effect of the desobliteration procedure on survival. The subgroups were as follows: (1) control group—no sign of shunt occlusion and (2) shunt occlusion group—desobliteration was performed. Survival times were calculated from the last operation, from the date of shunt implantation in Group 1, and from the date of desobliteration in Group 2.

**Figure 1:**
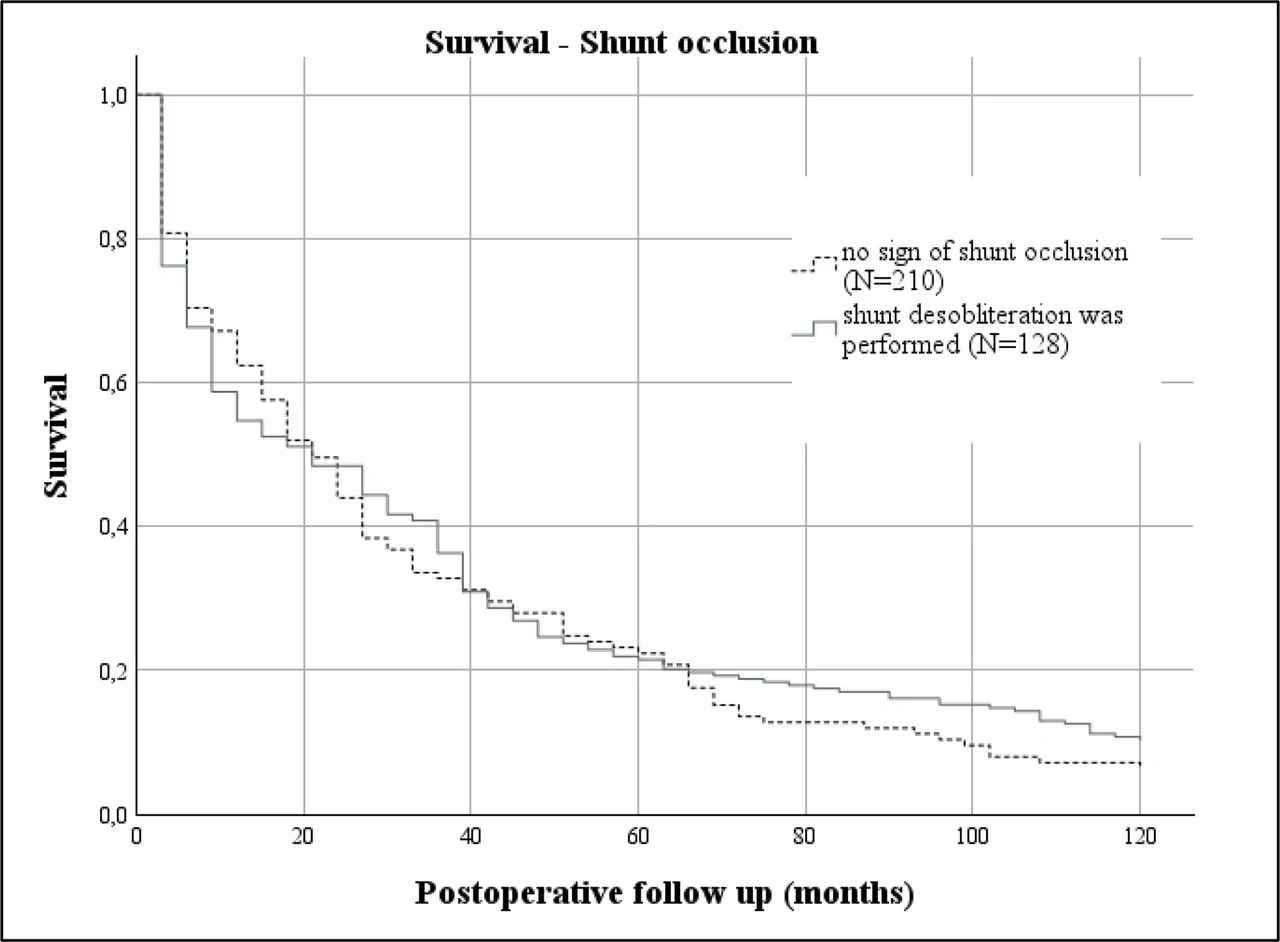
Shunt occlusion and survival. Cox regression analyses (HR=0.89, 95% CI 0.71 to 1.11, p=0.304).

There was no difference in the severity of liver disease (as measured by Child-Pugh and MELD scores) or operative mortality between the subgroups. The mean survival time of the shunt occlusion group was recorded at 35 months (95% CI 28 to 42 months), with a median survival of 20 months (95% CI 13 to 26 months). The mean survival time of the control group was recorded at 43 months (95% CI 35 to 51 months), with a median survival of 20 months (95% CI 11 to 30 months). As shown in figure 1, there was no significant difference in survival between the subgroups (HR=0.89, 95% CI 0.71 to 1.11, p=0.304). The 1-year and 5-year survival rates were 73% and 26%, respectively, which slightly exceeded the survival rates of the entire study population.

In the strictly selected population, after 2006, where LT was contraindicated, we studied 82 patients. Patient characteristics are summarized in table 4. The mean survival time was 33 months (95% CI 25 to 40 months), with a median survival of 20 months (95% CI 5 to 36 months). The survival rate deteriorated compared with patients before 2006, but the difference was not significant (HR=1.036, 95% CI 0.80 to 1.33, p=0.782). The 1-year and 5-year survival rates were 53% and 20%, respectively.

**Table 4:** Patient characteristics—changes after 2006

	Gender		Age (year)	
	Male	Female	≤ 59	≥ 60
Before 2006	67%	32%	72%	28%
After 2006	78%	22%	61%	39%

	Child-Pugh score	
	Child-Pugh B	Child-Pugh C
Before 2006	75%	25%
After 2006	60%	40%

	MELD score			
	≤9	10–19	20–29	30–39
Before 2006	26%	52%	22%	0
After 2006	18%	48%	34%	0

MELD, model for end-stage liver disease.

Long-term survival rate of the different subgroups is summarized in table 5.

**Table 5:** Long-term survival after PVS implantation

	1-year survival	5-year survival
Total study population	63%	23%
MELD score		
<9	75%	42%
10–19	71%	25%
20–29	43%	8%
Child-Pugh score		
B	74%	30%
C	40%	12%
Shunt correction	74%	26%
After 2006	52%	18%

MELD, model for end-stage liver disease; PVS, peritoneovenous shunt.

Following the univariate Cox regression analyses, a multivariate Cox proportional hazards model was applied to evaluate the independent effect of covariates on survival. The overall model was highly significant (Omnibus test of model coefficients, p<0.001), confirming that the included predictors together contributed significantly to survival.

Among the variables tested, MELD score remained the strongest independent prognostic factor (global p<0.001). Patients in the highest MELD category (20–29) had a 2.7-fold increased risk of death compared with the reference group (HR=2.68, 95% CI 1.62 to 4.42, p<0.001). The intermediate MELD category showed a non-significant trend toward increased mortality (HR=1.24, 95% CI 0.94 to 1.63, p=0.13).

Other factors, including Child-Pugh class (HR=1.24, 95% CI 0.82 to 1.88, p=0.31), repeated surgery (HR=1.65, 95% CI 0.92 to 2.97, p=0.09), and preoperative variceal sclerotherapy (HR=0.77, 95% CI 0.55 to 1.07, p=0.12), were not independently associated with survival in the multivariate model, although some showed borderline significance.

Taken together, the multivariate Cox regression confirmed that the MELD score is an independent and robust predictor of postoperative survival, whereas Child-Pugh classification and perioperative factors lost significance when analyzed simultaneously.

## Discussion

To understand the mechanism and beneficial effects of PVS, it is necessary to review the pathophysiology of ascites formation. In liver cirrhosis, histological remodeling causes an increase in intrahepatic resistance, which is the initial step of the complex hemodynamic changes. According to Ohm’s law, an increase in intrahepatic vascular resistance leads to portal hypertension. Portal hypertension is associated with the development of a peripheral, mainly splanchnic arterial vasodilation.^15 16^ The result of hemodynamical changes is the relative arterial underfilling, which leads to the release of vasoconstrictors and activation of sodium-retaining neurohumoral mechanisms with subsequent water and sodium retention.^17^

The PVS facilitates the drainage of ascitic fluid from the high-pressure peritoneal cavity to the low-pressure central venous system. The delivery of the ascitic fluid to the circulation improves the vicious circle emphasized above. Reinfusion of ascites reduces systemic hypovolemia and abdominal pressure, improves liver and kidney circulation, oxygenation of liver cells, and helps to restore energetic balance. Notably, the PVS not only relieves ascites and improves the quality of life but it may also allow the liver to recover spontaneously, as seen in several cases.^18^ According to the main guidelines, ascites in liver cirrhosis represents the end state of the disease. The basis of this opinion is that the histological process of liver cirrhosis is irreversible. However, this is not the case; with the successful treatment of ascites, liver function also improves, and regression of hepatic fibrosis is proven by several studies.^19^

Our retrospective study of a large number of PVS implantations shows that this procedure is an effective treatment for refractory ascites, particularly in cases of alcoholic cirrhosis. The mean survival of the study population was 43 months, with a median survival of 25 months, which is a remarkable figure in this disease.

If palliative treatment options are compared, according to the most recent meta-analysis in the field, including 77 studies^20^: 1-year survival rate in patients with TIPS was 67%, with large volume paracentesis 55%, with alfapump 56%, and with PVS 56%. 1-year survival rate in our study population was 63%, which does not explain the highly restricted place of PVS in treatment guidelines. The pathophysiological basis of PVS implantation is able to break the vicious circle leading to the development of ascites. Survival results do not lag behind other palliative procedures in refractory ascites. In a unique way among palliative interventions, after PVS implantation, liver function has a chance to improve, although usually, ascites does not disappear completely, and a steady state is reached. In 8% of the cases, complete regression of ascites is observed.

The frequency of shunt occlusion is the most important argument against PVS implantation. According to our data, if the desobliteration procedure is performed, the survival rate does not deteriorate, and the proportion of long-term survivors is remarkable. In our study, shunt occlusion was not considered as a failure of the therapeutic modality because we can treat it with a low-risk second surgery. Desobliteration after shunt occlusion is usually successful, and the mortality and morbidity did not differ after the first and second surgeries. Therefore, the frequency of shunt occlusion should not be considered as a contraindication to this modality.

Another important contraindication for PVS is frequent disseminated intravascular coagulation (DIC) during the postoperative period. In our study, DIC was detected in only one case. Our better results can be explained by modifications of the surgical technique. During surgery, a significant part of the ascites fluid is replaced with Ringer’s lactate to reduce the incidence of postoperative coagulopathy. Another modification is the use of anticoagulant treatment. At the end of the operation, anticoagulant treatment was started with intravenous unfractionated heparin, and then oral anticoagulant treatment was introduced. To reduce bleeding complications, upper gastrointestinal panendoscopy was required. In the case of grades 3–4 esophageal varices, we performed sclerotherapy 2 weeks prior to shunt implantation. The beneficial effect of continuous anticoagulant treatment on shunt patency could not be examined accurately due to the study design, but the incidence of bleeding complications was low. The routine use of anticoagulant therapy requires further investigation.

Our study focuses on the treatment of refractory ascites as a complication of alcoholic liver disease (ALD).^21^ The development of refractory ascites is associated with significantly reduced survival life expectancy; therefore, LT should be considered as a potential treatment option.^8^ Referral for LT and the pre-LT evaluation are decisive steps in patient care.

The place of ALD among the indications for LT has been a contentious issue in recent decades. End-stage ALD patients commonly exhibit a high prevalence of multisystem alcohol-related changes; neurological, cardiopulmonary, hematological, gastrointestinal, musculoskeletal, or psychiatric comorbidities are frequent. Psychological evaluation and assessment of the patient’s social support network are also key parts of the examination. Due to these factors, relatively fewer ALD patients are placed on an LT waiting list. According to a recent study in the USA on end-stage ALD patients who met the indication for LT, only 4% were on the waiting list and 1.2% underwent LT.^22 23^ Our data after 2006 show the efficacy of PVS implantation on a strictly selected patient population. During this period, all patients had contraindications for TIPS implantation and were not eligible for LT. However, the survival rate did not deteriorate significantly. In particular, this selected group of end-stage ALD patients with a poor prognosis can benefit from PVS implantation.

Should PVS implantation be avoided in the treatment of refractory ascites? The answer is definitely not. According to our data, PVS may be a reasonable option in selected patients, particularly where other options like TIPS or LT are not feasible.

## Data Availability

Data are available on reasonable request. The clinical data used are available in SPSS tables. Personal data are not identifiable.
